# Predicting responses to chemotherapy in the context that matters - the patient

**DOI:** 10.1080/23723556.2015.1057315

**Published:** 2015-06-10

**Authors:** Alicia Moreno-Gonzalez, James M. Olson, Richard A. Klinghoffer

**Affiliations:** a Presage Biosciences, Seattle, WA, USA; b Clinical Research Division, Fred Hutchinson Cancer Research Center, Seattle, WA, USA; c Department of Pediatrics, University of Washington, Seattle, WA, USA; d Seattle Children's Hospital, Seattle, WA, USA

**Keywords:** Cancer, clinical drug development, human lymphoma, *in vivo*, microinjection, preclinical, tumor response, tumor microenvironment

## Abstract

Guided by the belief that the most important setting for understanding tumor response to drugs is the human patient, we developed a technology called CIVO. CIVO enables analysis of up to 8 therapies simultaneously in a patient's tumor, without inducing systemic toxicity and while maintaining the integrity of the native tumor microenvironment.

## Abbreviations and acronyms

CC3: cleaved caspase 3
DNA-PK: DNA-dependent protein kinase
γH2AX: phospho-histone H2AX
pHH3: phospho-histone H3
mTOR: mammalian target of rapamycin
mTORC: mTOR complex.

## 

The troubling statistic that 9 out of 10 new cancer drugs entering clinical trials fail to provide benefit to patients^1^ indicates that there is a disconnect between tumor responses observed in translational models of cancer and anticancer efficacy in the clinic. This creates a fundamental problem for cancer drug development. Commonly used cell line models of cancer more often than not lack critical components of the tumor microenvironment. Cell lines grafted into immune-deficient mice are often used to recapitulate some aspects of *in vivo* tumor growth, but important components of the native tumor stroma and the host immune system are clearly compromised. Patient-derived xenograft models have been lauded as a more “clinically relevant” alternative, but their utility is limited by loss of native stroma upon passage, as well as lack of a complete immune system.^2^ Genetic analysis of human patient tumor samples has been used to identify genetic anomalies that drive resistance to cytotoxic agents and targeted inhibitors. While performed in clinically relevant samples, the information derived is best utilized for generating hypotheses regarding effective combination strategies, and not for functionally evaluating them.^3-7^ Ultimately, improving drug selection for cancer patients depends on evaluating drug sensitivity in the patient's own tumor with their own intact tumor microenvironment. To address this problem our team set out to develop a technology that could demonstrate drug activity without the toxicities associated with typical clinical exposures and in the setting where they need to work: the patient's own tumor.

**Figure 1.**
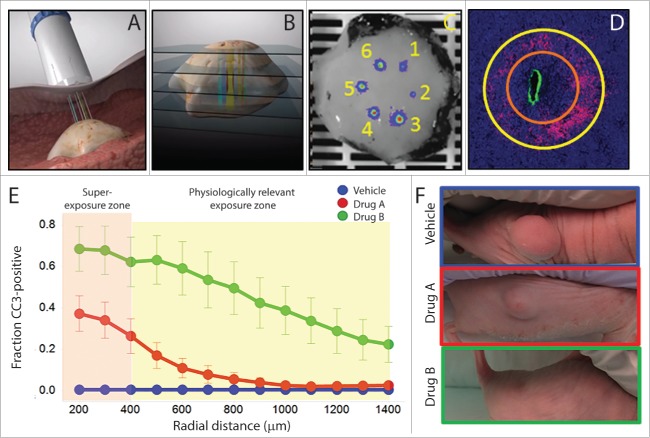
The CIVO tumor microinjection platform – localized responses predict systemic effects of chemotherapy. (A-B) The CIVO platform consists of a handheld array of up to 8 needles capable of simultaneously penetrating subcutaneous solid tumors and delivering easily identifiable column-like tracks of drug spanning the z axis of the tumor. (C) A chemically inert fluorescent tracking marker is co-injected through each needle to indicate each injection site within the tumor. (D) Tumor responses are assessed following tumor resection via histologic staining of tumor cross-sections sampled perpendicular to each injection column. These are quantified by an automated image-based analysis package that outlines radial zones where cells are super-exposed to drug (< 400 μm – orange ring) and those where cells are exposed to physiologically-relevant drug levels (> 400 μm - yellow ring). (E-F) The fraction of biomarker-positive cells (cleaved caspase-3 [CC3]) is plotted as a function of radial distance from the injection site and localized tumor responses are compared to tumor growth inhibition after systemic treatment.

The technology we developed, called CIVO, enables simultaneous evaluation of multiple drugs and drug combinations directly in a patient's living tumor, capturing the influence of the native tumor microenvironment and the patient's own immune system. CIVO consists of a hand-held microinjection device that delivers minute doses of up to 8 drugs or drug combinations directly into a solid tumor, leaving an easily identifiable column-like track that spans the depth of the tumor. The device is complemented by an automated quantitative image-based analysis package, called CIVO Analyzer, which measures the response of tens of thousands of individual cancer cells and surrounding stromal cells to drug exposure (Fig. 1 A-D). Importantly, because the levels of drug injected are miniscule, typically 1/100 of a normal systemically delivered dose, investigation of drug efficacy occurs without inducing toxic side effects associated with typical clinical exposures. In a recent publication^8^ we demonstrated several key steps toward using CIVO to perform comparative drug efficacy studies in the human oncology clinic.

Starting with well-characterized chemotherapy agents used to treat lymphomas, we found that by engineering our device to deliver a constant microliter volume per millimeter of tumor as needles are retracted, a spatially confined column of drug is left behind that spans the z-axis of the tumor. The injected drugs diffuse into gradients surrounding the initial injection site, allowing the use of distance from the epicenter of the injection site as a surrogate for drug concentration. Furthermore, by generating drug concentration–response curves for each and every injection performed, we can not only quantify drug response from cells that were exposed to physiologically-relevant drug levels (> 400 μm zone) but also interrogate cells that were super-exposed to drug (< 400 μm zone) (Fig. 1 D-E). This was confirmed by looking directly at drug distribution with radiolabeled compounds and also observed with biomarkers of tumor cell responses such as apoptosis (with cleaved caspase-3 [CC3]), DNA damage (phospho-histone H2AX [γH2AX], and mitotic arrest (phospho-histone H3 [pHH3]). All drugs induced the expected mechanism of action-specific tumor cell responses around the sites of injection that were easily observable upon histologic staining and quantified with our CIVO Analyzer.

Next, we found that the localized responses induced by drug microinjection that were observable within 24 hours after injection predicted long-term responses as measured by tumor growth inhibition following repeated systemic delivery over a period of 4 weeks. We demonstrated predictive value in both positive and negative directions and also showed that we could detect tumor context-specific responses to different drugs. This was observed with our test set of chemotherapy agents (vincristine, doxorubicin, mafosfamide/cyclophosphamide, and prednisolone/prednisone), and ultimately with a novel dual mammalian target of rapamycin (mTOR)/DNA-dependent protein kinase (DNA-PK) inhibitor CC-115 (an inhibitor of mTOR complex 1 [mTORC1], mTOR complex 2 [mTORC2], and DNA-PK).

Arguably most importantly, we established the feasibility of translating our technology into the oncology clinic. CIVO was pilot tested in a feasibility study to introduce vincristine to enlarged cancerous lymph nodes of 4 human patients enrolled at the Seattle Cancer Care Alliance. Similar to what we observed in the preclinical setting, microinjection of vincristine induced a localized area of tumor cell response. Equally important, no adverse events greater than grade 1 were reported and patient feedback regarding the experience was positive, with mild to no discomfort reported.

In the next year we expect to expand clinical investigation with CIVO to other solid tumor indications including sarcoma, melanoma, breast cancer, and head and neck cancers. Because CIVO can be employed in the clinical setting, we are beginning to interrogate not only cancer cell-specific responses to drugs, but also microenvironmental responses. As an example, we are beginning to observe significant drug-specific immune cell responses in our studies on canine patients with sarcoma. Furthermore, we are beginning to isolate and characterize the small population of cells within the super-exposure zone that survive high drug concentrations. We believe that these cells are highly resistant clones that may represent the minimal residual disease that often exists following the completion of chemotherapy. These resistant cells may lead to disease relapse. By proactively characterizing these cells, we intend to investigate drug combinations that attack key molecular drivers of resistance. Our goal is to recognize effective combinations in the context of native heterogeneous tumors and, in doing so, identify therapeutic regimens that lead to more durable remissions for patients with difficult-to-treat cancers.
